# Temperature‐induced multi‐species cohort effects in sympatric snakes

**DOI:** 10.1002/ece3.8601

**Published:** 2022-02-07

**Authors:** Richard B. King

**Affiliations:** ^1^ Department of Biological Sciences and Institute for the Study of the Environment, Sustainability, and Energy Northern Illinois University DeKalb Illinois USA

**Keywords:** gestation, neonatal size, ovulation, parturition, *Storeria*, *Thamnophis*

## Abstract

In reptiles, reproductive maturity is often determined by size rather than age. Consequently, growth early in life may influence population dynamics through effects on generation time and survival to reproduction. Because reproductive phenology and pre‐ and post‐natal growth are temperature dependent, environmental conditions may induce multi‐species cohort effects on body size in sympatric reptiles. I present evidence of this using 10 years of neonatal size data for three sympatric viviparous snakes, Dekay's Brown snakes (*Storeria dekayi*), Red‐bellied Snakes (*S*. *occipitomaculata*), and Common Garter snakes (*Thamnophis sirtalis*). End‐of‐season neonatal size varied in parallel across species such that snout–vent length was 36%–61% greater and mass was 65%–223% greater in years when gestating females could achieve higher April–May (vs. June–July or August–September) operative temperatures. Thus, temperature had a larger impact during follicular enlargement and ovulation than during gestation or post‐natal growth. Multi‐species cohort effects like these may affect population dynamics and the magnitude of these effects may increase with climate change.

## Graphical abstract

Sympatric snakes exhibit parallel patterns of neonatal size variation among years. End‐of‐season neonatal size is greater in years when females achieve high operative temperatures for more time in spring during follicle enlargement and ovulation. Multi‐species cohort effects like these may affect population dynamics and increase with climate change.

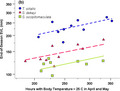

## 1 INTRODUCTION

For many reptiles, more rapid growth results in earlier maturity (Bronikowski & Arnold, 1999; Ford & Seigel, 1994; Frazer et al., 1993; Gibson & Hamilton, 1984). This means that growth early in life can influence population dynamics through effects on generation time and survival to reproduction (Cole, 1954; Gibbons et al., 1981; Oli & Dobson, 2003). Rapid neonatal growth can produce a “silver‐spoon effect” in which individuals that grow quickly early in life also experience higher growth rates later in life (Baron et al., 2010; Le Henanff et al., 2013; Madsen & Shine, 2000). Pre‐natal events can also influence post‐natal growth (Wapstra et al., 2010; While et al., 2009). For example, in Meadow Vipers (*Vipera ursinii ursinii*), earlier parturition is associated with greater offspring mass and body condition and faster post‐natal growth (Baron et al., 2010). Environmental temperature can influence both the timing of parturition (Blanchard & Blanchard, 1940; Cadby et al., 2010; Wapstra et al., 2010) and the rate of post‐natal growth (Adolph & Porter, 1996; Arnold & Peterson, 1989; Peterson et al., 1993), potentially inducing cohort effects with long‐term impacts on population dynamics (Beckerman et al., 2003; Lindstrom & Kokko, 2002; Wittmer et al., 2007). If environmental temperature has similar effects on multiple sympatric species, multi‐species cohort effects may result.

Here, I provide evidence that pre‐natal thermal conditions have parallel effects on end‐of‐season neonatal size in wild populations of three sympatric viviparous snakes, the Red‐bellied Snake (*Storeria occipitomaculata*), Dekay's Brown snake (*S*. *dekayi*), and the Common Garter snake (*Thamnophis sirtalis*). All three are colubrid snakes in the subfamily Natricinae (Pyron et al., 2013), are widely distributed and locally abundant across eastern (and western, *T*. *sirtalis*) North American (Powell et al., 2016), and have similar reproductive phenology. Mating mostly occurs in spring, followed by follicular enlargement and ovulation (Noble, 1937). At my study site in Illinois, enlarged follicles are first detectable by palpation in April and May. Gestation spans several months and parturition, as indicated by the appearance of neonates and post‐partum females, commences in late July or early August. Neonates lack yolk reserves at birth (Mack et al., 2017) but begin feeding soon after parturition and grow rapidly until cold weather brings about the cessation of aboveground activity (late September–mid‐October). Body size differs markedly among species, with *S*. *occipitomaculata* ranging from 67 to 284 mm snout–vent length (SVL) and 0.4–15.8 g, *S*. *dekayi* ranging from 76 to 378 mm SVL and 0.4–32.4 g, and *T*. *sirtalis* ranging from 115 to 780 mm SVL and 0.9–277.6 g at my study site. Diet also differs among species with *S*. *occipitomaculata* consuming almost exclusively slugs, *S*. *dekayi* consuming slugs, snails, and earthworms, and *T*. *sirtalis* consuming earthworms, amphibians, rodents, and birds (Virgin & King, 2019; personal observation).

## 2 METHODS

**FIGURE 1:**
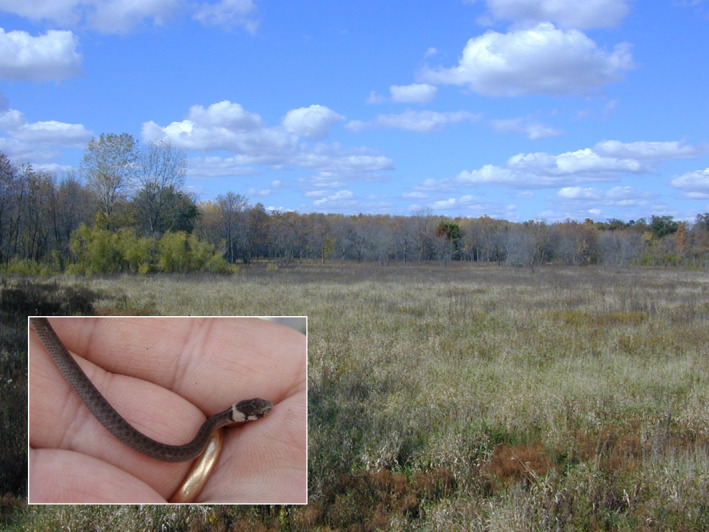
Late summer view of wet sedge meadow habitat at Potawatomi Woods Forest Preserve in northern DeKalb County, Illinois inhabited by *Storeria occipitomaculata*, *Storeria dekayi* (inset), and *Thamnophis sirtalis*. The *S*. *dekayi* shown was captured on 17 August 2017 and measured 77 mm snout–vent length and weighed 0.35 g. Photos by R. King

I conducted a capture–mark–recapture study of *S*. *occipitomaculata*, *S*. *dekayi*, and *T*. *sirtalis* at Potawatomi Woods Forest Preserve in northern DeKalb County, Illinois (42.4051 N, −88.8635 W) between April 2009 and October 2018. Fieldwork was focused in a wet sedge meadow and adjacent old field (approximately 5 ha; Figure 1). To facilitate snake detection, I placed 33–41 artificial cover objects (used rubber conveyor belt measuring ca. 60 × 90 × 1 cm) 15–20 m apart in an irregular grid. I checked artificial cover objects approximately weekly and captured snakes by hand. I classified snakes by species and sex and measured snout–vent length (SVL) using a cloth tape and mass using an electronic balance (Fitch, 1987). Snakes were individually marked by clipping ventral scales (using 3.5‐× magnification) and released where captured, usually within 10 min.

**FIGURE 2:**
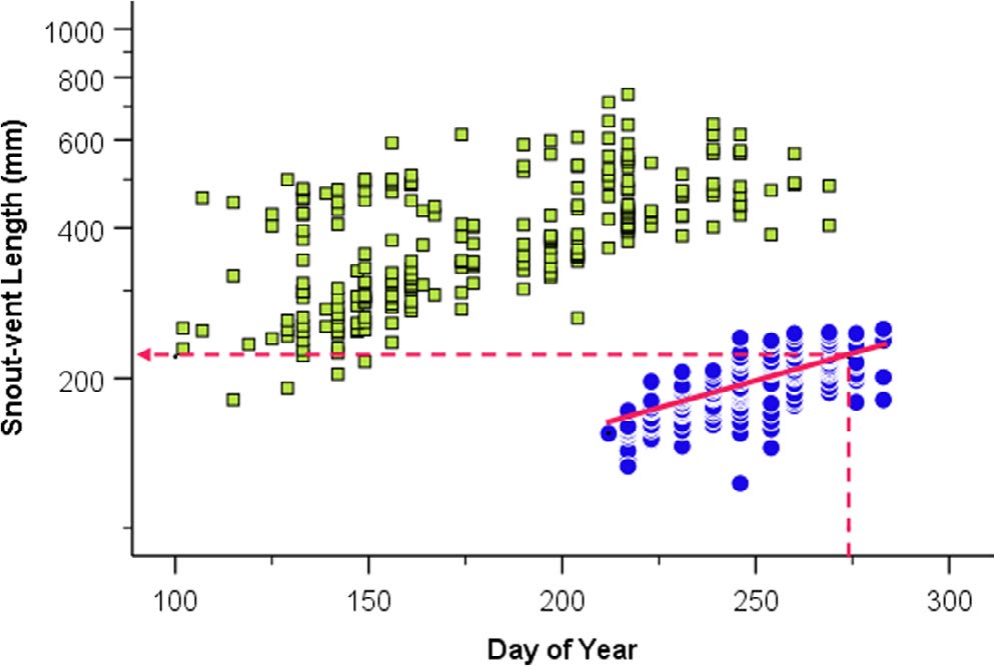
Relationship between snout–vent length and day of year for *Thamnophis sirtalis* in 2014. Neonates (filled circles) appear as a size class distant from older snakes (open squares) whose growth trajectory was estimated by regression (solid line). Dashed lines show estimation of end‐of‐season snout–vent length, the expected snout–vent length on day of year 274

I identified neonates (animals captured prior to their first hibernation) as a distinct age class by plotting SVL against day of year (DOY) separately for each year and species (an example is shown in Figure 2). For each species, I used analysis of covariance with neonatal SVL or neonatal mass as dependent variable, year as factor, DOY as covariate, and including the year‐by‐DOY interaction, to generate equations relating SVL and mass to DOY. Prior to analysis, I transformed SVL and mass by adding 1 and computing natural logarithms to linearize relationships and homogenize variances (Zar, 2010; analyses of untransformed data yielded virtually identical results). To compare year‐to‐year variation in neonatal growth, I computed the expected SVL and mass on October 1, the approximate end of the active season, for each year and species combination.

To identify possible temperature‐related causes of year‐to‐year variation in end‐of‐season SVL and mass, I estimated operative body temperatures using the hindcaster feature of NicheMapR (microclimate model with gridMET USA meteorological grids and ectotherm model, http://bioforecasts.science.unimelb.edu.au/app_direct/ectotherm_usa/; Kearney, 2020; Kearney & Porter, 2017, 2020). Operative temperatures were estimated separately for three periods corresponding to follicular enlargement and ovulation (April–May), gestation (June–July), and post‐natal growth (August–September). For April–May and June–July, I set animal mass to the mean mass of gravid females at my study site (*S*. *occipitomaculata* = 9.7 g, *S*. *dekayi* = 17.5 g, and *T*. *sirtalis* = 76.8 g); for August–September, I set animal mass to the mean mass of neonates at my study site (*S*. *occipitomaculata* = 1.0 g, *S*. *dekayi* = 1.5 g, and *T*. *sirtalis* = 3.5 g; see Appendix 1 for other model settings). For each species, year, and period, I computed the number of hours that body temperature exceeded 25°C, the temperature at which natricinae digestive rate, crawling speed, oxygen consumption, and tongue flick rate reach ca. 50% of their maxima and above which oxygen consumption increases rapidly from baseline (Stevenson et al., 1985). At Long Point, Ontario, a site similar in latitude and elevation to my study site, more than 90% of active *T*. *sirtalis* body temperatures exceeded 25°C (Figure 1 in Gibson & Falls, 1979). The thermal biology of *Storeria* is less well known but body temperatures in excess of 25°C occurred throughout the active season at a site in northwestern PA (Gray, 2014) and locomotor performance increased from 10 to 20 to 30°C (Gerald & Claussen, 2007). I used analysis of covariance with species as a factor to test whether end‐of‐season SVL or mass covaried with hours >25°C in April–May, June–July, or August–September. I first tested for a significant factor‐by‐covariate interaction to determine if the slope of the relationship between end‐of‐season SVL or mass and hours >25°C differed among species. When no such interaction was detected (Results), main effects were tested in follow‐up analyses of covariance with the factor‐by‐covariate interaction omitted. I generated estimates of effect size (partial *η* ^2^; the proportion of variation in end‐of‐season SVL or mass explained by hours >25°C after removing variation attributable to species; Richardson, 2011) to assess the magnitude of each period's influence. For comparison, I computed mean April–May, June–July, and August–September air temperatures from daily temperature data downloaded from https://prism.oregonstate.edu/. IBM SPSS Statistics Version 26 (Armonk, New York) was used for analysis with *α* = .05.

## 3 RESULTS

**FIGURE 3:**
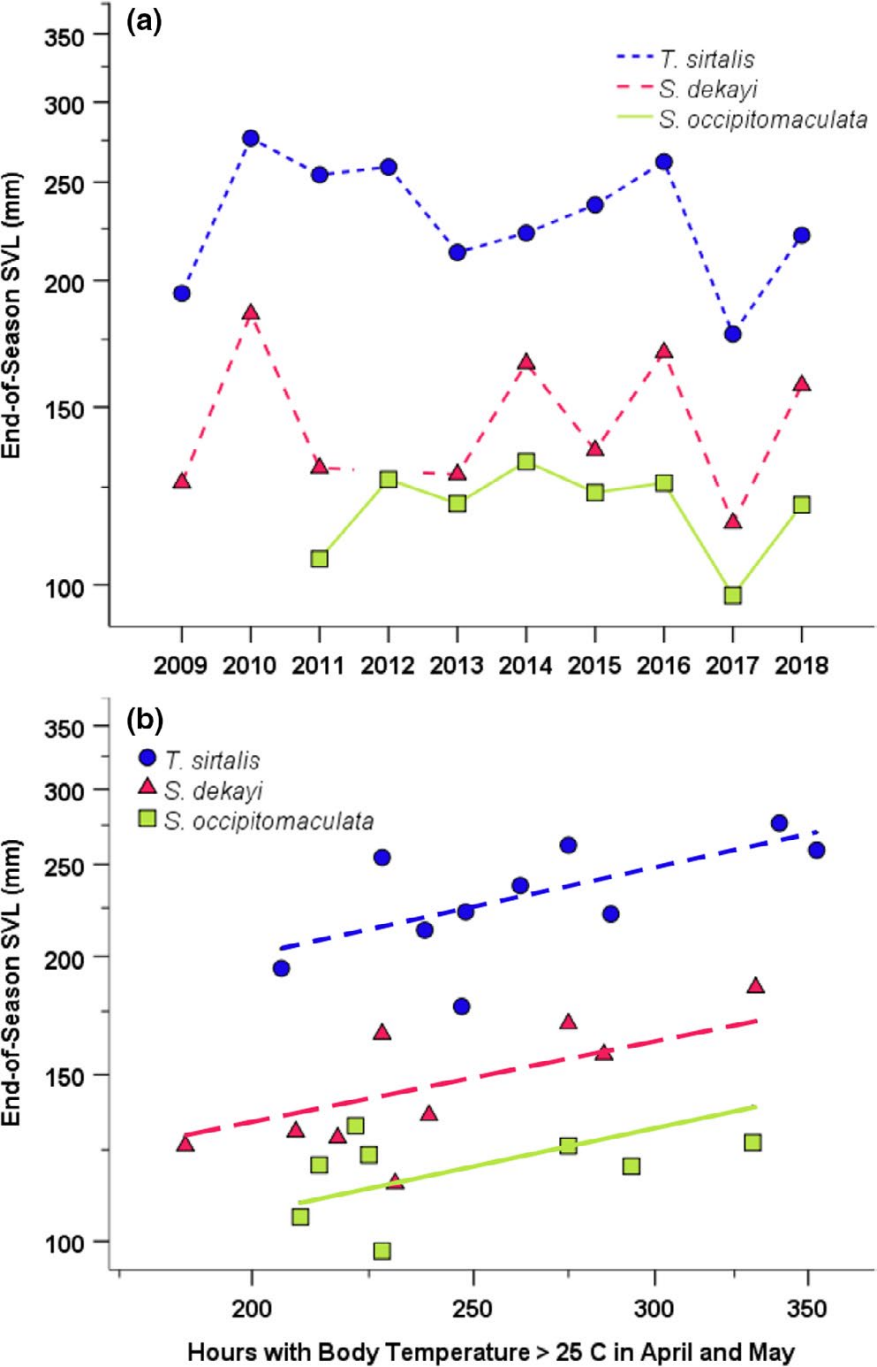
Yearly variation in end‐of‐season neonatal SVL (a) and relationship between end‐of‐season neonatal SVL and the estimated hours that gravid female body temperature exceeded 25°C in April and May (b) for *Storeria occipitomaculata* (squares), *Storeria dekayi* (triangles), and *Thamnophis sirtalis* (circles) from 2009 to 2018. Small sample size precluded computing end‐of‐season SVL and mass for *S*. *dekayi* in 2012 and for *S*. *occipitomaculata* in 2009 and 2010

Neonate captures numbered 10–64 per year for *S*. *occipitomaculata* (total *n* = 269 after excluding 2009 and 2010 due to small sample size), 10–106 per year for *S*. *dekayi* (total *n* = 437 after excluding 2012 due to small sample size), and 21–193 per year for *T*. *sirtalis* (total *n* = 988). Analysis of covariance revealed that in each species, the relationship between SVL and DOY differed in slope among years as indicated by a significant year‐by‐DOY interaction (*S*. *occipitomaculata*: *F*_7,253_ = 7.27, *p* < .001; *S*. *dekayi*: *F*_8,418_ = 4.65, *p* < .001; and *T*. *sirtalis*: *F*_9,962_ = 7.15, *p* < .001; Appendix 2). Similarly, the relationship between mass and DOY differed in slope among years (*S*. *occipitomaculata*: *F*_7,251_ = 5.23, *p* < .001; *S*. *dekayi*: *F*_8,419_ = 5.67, *p* < .001; and *T*. *sirtalis*: *F*_9,965_ = 6.45, *p* < .001; Appendix 2). End‐of‐season SVL and mass varied in parallel among species (SVL: intraclass correlation = 0.73, 95% confidence limits = 0.37, 0.94; mass: intraclass correlation = 0.53, 95% confidence limits = 0.02, 0.89; Zar, 2010, pp 411–414), were greatest in 2010 and 2016, and were least in 2009 and 2017 (Table 1, Figure 3a).

**TABLE 1:** Number of hours that body temperatures are expected to exceed 25°C in April–May, June–July, and August–September and end‐of‐season SVL and mass of neonatal *Storeria occipitomaculata*, *Storeria dekayi*, and *Thamnophis sirtalis* at Potawatomi Woods Forest Preserve, DeKalb County, Illinois in 2009–2018

Year	Body temperature hours >25°C			End‐of‐Season SVL (mm)	End‐of‐Season Mass (g)
	April–May	June–July	August–September		
*S. occipitomaculata*					
2009	183	543	427		
2010	324	693	495		
2011	210	707	431	106.1	0.8
2012	331	733	511	127.2	1.3
2013	214	623	527	120.4	1.2
2014	222	576	455	132.5	1.3
2015	225	627	508	123.5	1.1
2016	275	692	513	126.2	1.3
2017	228	664	492	97.6	0.8
2018	293	658	494	120.0	1.1
*S. dekayi*					
2009	187	550	432	126.3	1.2
2010	332	699	506	185.5	2.7
2011	209	721	435	130.6	1.6
2012	336	739	519		
2013	218	627	529	128.7	1.6
2014	228	595	463	165.7	2.4
2015	239	631	513	135.9	1.6
2016	275	696	524	169.9	2.9
2017	231	666	495	115.1	1.2
2018	285	663	501	157.6	2.3
*T. sirtalis*					
2009	206	571	454	194.3	4.4
2010	340	710	516	276.3	10.4
2011	228	712	442	254.3	9.2
2012	353	753	531	258.8	10.1
2013	238	642	538	213.2	5.7
2014	248	612	473	222.8	5.5
2015	262	639	529	237.5	6.2
2016	275	704	540	262.0	9.9
2017	247	677	506	177.1	3.2
2018	287	674	509	221.7	6.0

Note

Small sample size precluded computing end‐of‐season snout–vent length and mass for *S. dekayi* in 2012 and *S. occipitomaculata* in 2009 and 2010. Values shown for SVL and mass are back‐transformed from the regression of ln(SVL+1) and ln(mass+1) on DOY (Appendix 2) for DOY = 274.

Depending on species, body temperature was predicted to exceed 25°C for an average of 250.5–268.4 h in April–May (range = 183–253), 651.6–669.4 h in June–July (range = 543–753), and 485.3–503.8 h in August–September (range = 427–540; Table 1). Tests for a difference in slope among species in the relationship between end‐of‐season SVL and mass to hours >25°C were consistently non‐significant (April–May – SVL: *F*_2,21_ = 1.249, *p* = .304, mass: *F*_2,21_ = 1.457, *p* = .255; June–July – SVL: *F*_2,21_ = 1.482, *p* = .250, mass: *F*_2,21_ = 3.087, *p* = .067; August–September – SVL: *F*_2,21_ = 0.015, *p* = .985, mass: *F*_2,21_ = 0.021, *p* = .979). In subsequent analyses with the factor‐by‐covariate interaction omitted, species had consistently significant effects on end‐of‐season SVL and mass (*F*_2,23_ = 51.969–85.889, *p* < .001), April–May hours >25°C had significant effects on end‐of‐season SVL and mass (SVL: *F*_1,23_ = 15.053, *p* = .001; mass: *F*_1,23_ = 12.795, *p* = .002; Figure 3b), June–July hours >25°C had a no significant effect on end‐of‐season SVL but did have a significant effect on mass (SVL: *F*_1,23_ = 2.245, *p* = .148; mass: *F*_1,23_ = 5.875, *p* = .024), and August–September hours >25°C had no significant effect on end‐of‐season SVL or mass (SVL: *F*_1,23_ = 1.654, *p* = .210; mass: *F*_1,23_ = 2.104, *p* = .160). Estimated effect sizes indicated that April–May hours >25°C had the largest effect on end‐of‐season SVL and mass (SVL: partial *η* ^2^ = 0.40; mass: partial *η* ^2^ = 0.36), June–July hours >25 had small to medium effects (SVL: partial *η* ^2^ = 0.09; mass: partial *η* ^2^ = 0.24), and August–September hours >25 had only small effects (SVL: partial *η* ^2^ = 0.07; mass: partial *η* ^2^ = 0.08). Similar results were obtained using thresholds of 10°C and 20°C except that August–September hours >20°C had a significant effect on end‐of‐season SVL and mass (SVL: *F*_1,23_ = 4.909, *p* = .037, partial *η* ^2^ = 0.18; mass: *F*_1,23_ = 5.339, *p* = .030, partial *η* ^2^ = 0.19). The duration of time that body temperature was predicted to exceed 25°C was positively, but imperfectly, correlated with mean air temperature (April–May: *r* ^2^ = .66, .73, and .79 for *S*. *occipitomaculata*, *S*. *dekayi*, and *T*. *sirtalis*, respectively; June–July: *r* ^2^ = .97, .98, and .98: August–September: *r* ^2^ = .66, .70, and .70; all *p* < .05).

## 4 DISCUSSION

Sympatric *S*. *dekayi*, *S*. *occipitomaculata*, and *T*. *sirtalis* showed parallel patterns of variation in neonatal size across 10 years such that end‐of‐season SVL was 36%–61% greater and end‐of‐season mass was 65%–223% greater in years with maximal size relative to years with minimal size. Furthermore, end‐of‐season SVL and mass were associated with the amount of time that gravid females could achieve April–May body temperatures >25°C. This result suggests that the rate follicular enlargement and timing of ovulation had especially large impacts on neonate size. Variation in the amount of time that females could achieve June–July body temperatures >25°C (gestation and parturition) or the amount of time that neonates could maintain August–September temperatures >25°C (post‐natal growth) had less impact. Of these three periods, the amount of time that snakes could achieve body temperatures >25°C was least for April–May (averaging ca. 260 h vs. 660 h in June–July and 490 h in August–September) and had the largest among‐year coefficient of variation (23%–27% depending on species vs. 9%–11% in June–July and 8%–9% in August–September). Behavioral thermoregulation may allow gestating females to achieve their preferred body temperatures more easily in June–July when ambient temperatures are high than in April–May when ambient temperatures are lower (Huey et al., 1989; Peterson, 1987). Possibly, the small size of neonates limits their thermoregulatory ability during August–September (Bittner et al., 2002). Alternatively, the thermal dependence of physiological processes in neonates may differ from that of adults as suggested by the significant association of end‐of‐season SVL and mass with August–September hours >20°C but not >25°C.

Experimental manipulations of environmental temperatures in semi‐natural enclosures could provide more rigorous tests of thermal effects on neonatal size (Blouin‐Demers et al., 2000; Le Henanff et al., 2013; Lourdais et al., 2004). For example, in a multi‐year study of *T*. *sirtalis* maintained in outdoor enclosures, Blanchard and Blanchard (1940) found that parturition dates were accelerated 4.5 days per °F increase in mean May–July temperature (=8.1 days per °C). Given that April–July temperatures differed by 3.3°C among years at my study site (https://prism.oregonstate.edu/), their results suggest that parturition dates might vary ca. 25 days among years, shortening or extending the time available for post‐natal growth accordingly. Unfortunately, accurate estimates of the timing of ovulation and parturition are difficult to obtain and are likely to vary among individuals in the field (but see Sparkman et al., 2018).

Cohort effects at my study site were of sufficient magnitude to accelerate attainment of reproductive maturity in at least some individuals during warm years. For example, in 2010, the end‐of‐season SVL of *S*. *dekayi* neonates (185.5 mm) exceeded the minimum SVL of reproductively mature males (175 mm based on presence of sperm in cloacal smears, personal observation) and at least some neonatal males exceeded 175 mm in 2010, 2014, 2016, and 2018. Accelerated maturation promotes population growth by reducing the likelihood of mortality before reproductive maturity and by shorting generation time (Cole, 1954; Gibbons et al., 1981; Oli & Dobson, 2003). In addition, the larger size attained by neonates in warm years may result in increased survival (Jayne & Bennett, 1990) independent of age at reproductive maturity. Consequently, the cohort effects described here may generate temporal variation in population abundance, density, and size structure much like patterns of geographic variation attributed to differences in activity season (Adolph & Porter, 1996). Given the degree of dietary overlap among snake species at my study site, especially between *S*. *dekayi* and *S*. *occipitomaculata* (Virgin & King, 2019), temporal variation in abundance and density may affect competitive interactions among snake species and have top‐down and bottom‐up effects on their prey and predators. Additional data on the degree to which cohort effects persist beyond the neonatal life stage and the extent to which reproductive maturity is size‐ versus age‐dependent (Bronikowski & Arnold, 1999) would aid in evaluating their impact on population dynamics.

Cohort effects like those observed here are not unusual, having been documented in a wide range of plant and animal taxa (Lindstrom & Kokko, 2002 and citations therein). What is unusual, although not unexpected, is the occurrence of parallel cohort effects across multiple sympatric species. Because of their physiological dependence on environmental temperature (Huey, 1982), ectothermic vertebrates are likely candidates for exhibiting multi‐species cohort effects but similar patterns are anticipated in other taxa as a consequence of different shared environmental drivers (e.g., water availability in plants, Streng et al., 1986; beech masting in rodents, Wittmer et al., 2007; fire in grassland birds, Powell, 2006). Although analyses of cohort effects on single‐species population dynamics have been fruitful (Beckerman et al., 2003; Le Galliard et al., 2010; Lindstrom & Kokko, 2002; Wittmer et al., 2007), multi‐species cohort effects, with their potential impacts on competitive and predator–prey interactions, warrant further study (Huss et al., 2013). The more frequent occurrence of extreme weather events (IPCC, 2014) may result in even larger cohort effects than those observed here (Cadby et al., 2010; Lourdais et al., 2004). Equally interesting are situations where weather or other environmental drivers have contrasting effects on sympatric species due to differing ecological traits (e.g., Ma et al., 2018). For example, a hot year might have negative effects on diurnal or open‐habitat species, but positive effects on nocturnal or shade‐dwelling species, as has been suggested in the context of climate change (Huey et al., 2012; Paaijmans et al., 2013). The fact that cohort effects can arise from pre‐natal or pre‐ovulatory environmental conditions has the potential to magnify the impact of climate change on demography and life history.

## CONFLICT OF INTEREST

The author declares no conflict of interest.

## AUTHOR CONTRIBUTIONS

**Richard King:** Conceptualization (lead); data curation (lead); formal analysis (lead); investigation (lead); methodology (lead); project administration (lead); visualization (lead); writing – original draft (lead); writing – review and editing (lead).

## Data Availability

Data and NicheMapR R code are archived at https://doi.org/10.5061/dryad.tmpg4f50h.

## APPENDIX 1

Parameter values used to hindcast snake body temperatures using NicheMapR (http://bioforecasts.science.unimelb.edu.au/app_direct/ectotherm_usa/; Kearney & Porter, 2020). Parameters for which settings differ from default values are shown in bold

**Table:**

inputId	Value	variable_name	variable_unit
**latitude**	**42.1051**		**°**
**longitude**	**−88.8635**		**°**
**year**	**2009–2018**		
state	alive		
month to plot	all		
day to plot	all		
warm	0	°C warming	°C
Usrhyt	1	height, cm	cm
minshade	0	% shade	%
maxshade	90	maxshade (not used in soil apps)	%
slope	0	slope	°
aspect	0	aspect	°
hori	0	horizon	°
windfac	1	wind mult	–
REFL	20	% albedo	%
SLE	95	% emissivity	%
Thcond	2.5	thermal conductivity	W/(m K)
SpecHeat	870	heat capacity	J/(kg K)
Density	2.56	mineral density	kg/m3
BulkDensity	1.3	bulk density	kg/m3
cap	FALSE	organic surface layer?	–
soilgrids	FALSE	query soilgrids?	–
elevatr	FALSE	fine–scale topography?	–
res	30	resolution	m
pixels	100	pixels	#
clearsky	FALSE	run with clear sky?	–
state	alive	state	–
**Ww_g**	**1.0–76.8** ^a^	**mass**	**g**
**shape**	**cylinder**	**shape**	–
**shape_b**	**20**	**stretch**	–
diurn	TRUE	diurnal	–
nocturn	FALSE	nocturnal	–
**crepus**	**TRUE**	**crepuscular**	–
shadeseek	TRUE	seek shade	–
burrow	TRUE	burrow	–
**shdburrow**	**TRUE**	**burrow shade**	–
climb	FALSE	climb	–
mindepth	2.5 cm	min depth	cm
maxdepth	100 cm	max depth	cm
**T_F_min**	**20**	**min forage**	**°C**
T_F_max	35	max forage	°C
**T_B_min**	**10**	**basking**	**°C**
T_RB_min	10	leave retreat	°C
CT_min	5	critical min	°C
CT_max	40	critical max	°C
**T_pref**	**30**	**preferred**	**°C**
alpha	85	absorptivity	%
pct_wet	0.1	skin wetness	%
warmsig	0	heat signal	°C/h

^a^ Mass = 1.0, 1.5, and 3.6 for neonatal *Storeria occipitomaculata*, *Storeria dekayi*, and *Thamnophis sirtalis* and 9.7, 17.5, and 76.8 for gravid adult female *S*. *occipitomaculata*, *S*. *dekayi*, and *T*. *sirtalis*.

## APPENDIX 2

Regression coefficients relating ln(SVL+1) and ln(mass+1) to day of year in neonatal *Storeria occipitomaculata*, *Storeria dekayi*, and *Thamnophis sirtalis*, 2009–2018

**Table:**

	SVL		Mass	
	Intercept	Slope	Intercept	Slope
*S. occipitomaculata*				
2009				
2010				
2011	4.3059	0.0013	0.4206	0.0007
2012	3.9174	0.0034	−0.3993	0.0044
2013	2.8960	0.0069	−1.2866	0.0076
2014	2.9751	0.0070	−0.8501	0.0061
2015	4.0777	0.0027	−0.5638	0.0048
2016	3.0661	0.0065	−1.0149	0.0068
2017	4.9353	−0.0013	0.5480	0.0002
2018	3.4548	0.0049	−0.0920	0.0030
*S. dekayi*				
2009	3.2814	0.0057	−1.1966	0.0073
2010	3.8400	0.0051	1.1191	0.0007
2011	3.6233	0.0046	−0.5724	0.0055
2012				
2013	3.4901	0.0050	−1.2637	0.0081
2014	3.4807	0.0060	−0.7120	0.0071
2015	3.3576	0.0057	−1.6720	0.0096
2016	2.7460	0.0087	−2.2488	0.0132
2017	4.2435	0.0019	−0.1448	0.0034
2018	3.4133	0.0060	−1.3370	0.0093
*T. sirtalis*				
2009	3.7759	0.0055	−1.8513	0.0129
2010	3.4267	0.0080	−1.9957	0.0162
2011	3.3019	0.0082	−2.5524	0.0178
2012	4.0326	0.0056	−2.1186	0.0165
2013	3.8389	0.0056	−1.0607	0.0108
2014	3.8657	0.0056	−1.6214	0.0128
2015	3.8395	0.0060	−1.5390	0.0128
2016	3.5102	0.0075	−2.3028	0.0171
2017	4.7337	0.0016	0.3898	0.0038
2018	3.9557	0.0053	−1.4063	0.0122
